# Paralytic Phenomena in Simple Psychosis

**Published:** 1887-08

**Authors:** Lester Curtis


### CONCERNING PARALYTIC PHENOMENA IN SIMPLE PSYCHOSIS, ESPECIALLY MELANCHOLIA.

Report of a Clinic by Prof. Benswanger, of Jena, by Theodore Tichen. *Berliner Klinische Wochenschrift*, June 27, 1887.

THE writer refers to the fact that in a case of melancholia, or mania, physicians are accustomed to look for paralytic phenomena in the patient, and, if these occur, give an unfavorable prognosis, regarding the case as one of progressive paralysis.

But Seifert, in 1853, described cases "which in part, at least, belonged to pure mania and melancholia, with disturbance of the innervation of the iris. In two cases of simple mania there occurred a dilatation of the pupil on one side, which followed in its intensity all the variations of the psychic condition and disappeared for the first time on the occurrence of full recovery."

"Bailarger also emphasized the fact that *mania ambiticuse* with slight motor disturbances did not always lead to paralytic imbecility."

"Nasse confirmed Seifert's observations, and maintained that asymmetrical innervation of the facial nerve, hypoglossal nerve and the pupil, constant, as well as transitory, might not be of serious importance, and occurred in functional psychosis also, and, indeed, more frequently in such cases than in sane persons."

"Since then Tiggs alone has described similar paralytic phenomena, especially one-sided paresis of the face in simple melancholia, mania and dementia."

Later writers ignore the subject.

"I have observed in three cases of functional psychoses which were conditioned upon neither organic disease of the nervous system, nor upon hysteria, nor traumatism, etc., but occurred simply as phenomena attendant upon severe melancholia."

"In two cases the trouble was a stupor of exhaustion (*erschöpfungs-stupor*) and a melancholia which were associated with a moderate, but distinct, one-sided facial paresis, during rest as well as during active and mimetic motion."

"After perfect recovery there was perhaps still a slight difference between the two sides of the face while at rest, active innervation was perfectly symmetrical. The greater width of the fissure of the eye of the paretic side in this case had pointed to the participation of the eye facial. Asymmetry of the skull did not exist. Other disturbances of innervation were not noticeable."

"The third case, on account of its marked peculiarities, may be be given at length in the details of importance to the question."

"Kt., owner of a brick kiln, born in 1848, heavily burdened by inheritance, badly pulled down by the campaign of 1870–1, a sufferer from neither syphilis nor alcoholism. In the year 1875 he had, after a great fright, a severe attack of melancholia. He recovered after treatment in an asylum. He remained well for eleven years, when he had a slight attack of indigestion.

"In October, 1886, after the death of a child, he became ill again mentally, and, on account of severe depression, in which he attempted suicide, he was brought into this institution. Here, mentally, he was the picture of severe melancholia without the slightest intellectual defect; bodily, signs of premature senility, increase of the tendon and skin reflexes, in other respects almost no disturbance. In the progress of the mental affection there appeared paresis of the mouth facial (at rest as well as during active innervation), at one time on the right, at another on the left; its intensity corresponded in all respects with the degree of the melancholia. Meanwhile, after the institution of opium treatment (0.12 extr. opii. twice a day) severe retention of urine was added to the symptoms. With the cessation of the opium treatment, the bladder symptoms disappeared. But there occurred a constant dilatation of the right pupil, (with prompt reaction) and, in addition, complaints of tension and weight in the limbs.

"After about a month the psychic condition improved, the difference in the pupils was smaller, and the changing difference in the face no longer occurred.

" A relapse occurring, caused by the accidental visit of a friend, the difference between the pupils showed itself again quite plainly.

"About two and a half months after his reception he was dismissed ; the difference between the pupils was then no longer to be noticed, and motility, sensibility and intellect were intact."

" Cases like the two first mentioned are certainly more frequent than one would think. I might conjecture that here the functional disease brings forward only more prominently an already existing congenital deficiency in innervation. Hysterical symptoms also appear not seldom to be located by preference in such deficiently innervated nerve territories, or occur with greater intensity."

" In the third case which I may regard as melancholia, it is certainly possible to make the premature senility, next to the severe melancholia, answerable for the unusual paretic phenomena, without being willing to contend that it is self-evident that such a patient may have a special predisposition to a later progressive paralysis or senile dementia. Among paralytics especially, also, the degree of the paralysis is by no means in that close relation to the intensity of, for example, the of depression. The chief stress is in every case to be laid upon the absence of intellectual defects ; these are the first symptoms that establish the bad prognosis of anomalies of the passions and paralytic phenomena. At the same time a case like the above forms a transition from simple melancholia to curable *folie paralitique*; from the latter it is separated by the lack of the paralytic disturbance of speech."

" In connection with these cases attention may be drawn to still another paralytic phenomenon in functional psychoses, especially melancholia and the stupor of exhaustion ; this is a slight insufficiency of the internal recti muscles of the eye, for the most part on both sides, occasionally with the consequence of slight muscular asthenopia. It betrays itself by the loss of the accurate direction of both eyes toward the finger which has been held up when one eye is covered by the hand, the covered eye deviates slightly outwards. That the case behaves here purely as a phenomena attendant upon the psychosis is shown by this, that the insufficiency does not exist long before the psychosis and disappears upon recovery."

" Finally I may be allowed to give space to the following single observation concerning the innervation of the pupil, although, of course, here, as in Seifert's cases, it occurs more often as a result of irritation of the sympathetic than as a paralytic phenomenon."

" Brissand and Marie have of late repeatedly described cases in which the hypoglossal and facial nerves were involved in a hysterical hemiplegia. In their clinic there was a case of hysterical paranoia in which the left half of the body, also including the mouth facial was paretic. The tongue did not deviate, but there existed a *dilatation of the left pupil whose intensity went hand in hand with the hemiplegia*. The reactions of the pupil were well retained."

"In another case, of severe idea of constraint in a man sixty years of age, there existed a difference in the pupils for a few days, and occasionally also for a few weeks, in favor of the right eye, then it was transferred and the left pupil was again plainly wider. Now and then there was equality of the pupils. This variation has now been observed many times. Attacks of one-sided migrane (whose co-existence with melancholia, for example, is not rare) does not exist."

" Theoretically, the above paralytic phenomena arrange themselves as phenomena attendant upon simple psychoses (Freusberg). The foundation of such paralyses are congenital deficiency of innervation, fatigue and disturbance of the circulation of the cortical and sub-cortical centers (indirect influence of the sympathetic, here belongs case Kl.)."

" Practically the cases which have been cited seem to me to make it proper to be careful concerning the diagnostic realization of paralytic phenomena *when intellectual defects are wanting.* The latter is the first thing which can demonstrate positively organic disease of the brain. On the other hand, we know to-day that a typical progressive paralysis may develop from an inorganic psychosis, as, for example, hysteria. But those paralytic phenomena in the transient psychoses always possess value as warning symptoms."

LESTER CURTIS.

